# Dedifferentiated Liposarcoma of the Retroperitoneum with Extensive Leiomyosarcomatous Differentiation and *β*-Human Chorionic Gonadotropin Production

**DOI:** 10.1155/2008/658090

**Published:** 2008-03-12

**Authors:** Michael J. Russell, Frederick L. Flynt, Allyson L. Harroff, Oluwole Fadare

**Affiliations:** ^1^Department of Pathology, Wilford Hall Medical Center, Lackland Air Force Base, San Antonio, TX 78236, USA; ^2^Pathology Program, San Antonio Uniformed Services Health Education Consortium, San Antonio, TX 78236, USA; ^3^Department of Internal Medicine, Wilford Hall Medical Center, Lackland Air Force Base, TX 78236, USA; ^4^Department of Hematology/Oncology, Wilford Hall Medical Center, Lackland Air Force Base, San Antonio, TX 78236, USA; ^5^Department of Pathology, University of Texas Health Science Center at San Antonio, San Antonio, TX 78229, USA

## Abstract

Dedifferentiated liposarcomas may display a variety of “heterologous” lines of differentiation, including osseous, vascular, skeletal, and/or smooth muscular. There have been six previously reported examples of leiomyosarcomas associated with high levels of serum human chorionic gonadotropin (hCG) production, comprised of cases originating from the retroperitoneum, spermatic cord, small intestine, and uterus. This report describes the first example of a dedifferentiated liposarcoma that combined both of the aforementioned features: extensive heterologous (leiomyosarcomatous) differentiation and *β*-hCG production (maximum serum levels 1046 mIU/ml, reference <5 mIU/ml). The tumor, which originated in the retroperitoneum in the region of the right kidney, was rapidly progressive and ultimately fatal within three months of its diagnosis. In addition to characteristic morphologic features, lipogenic and smooth muscle differentiation were confirmed with immunohistochemical stains for MDM2 and smooth muscle actin, respectively. The tumor also displayed diffuse immunoreactivity for *β*-hCG in both primary and metastatic sites. This case further expands the clinicopathologic spectrum of lipogenic tumors.

## 1. INTRODUCTION

The term “tumor dedifferentiation” and its underlying concepts, as used by contemporary surgical pathologists, are based on the paradigm originally outlined by Dahlin and Beabout in low-grade chondrosarcomas [1]. Tumor dedifferentiation is characterized by the “emergence” of an undifferentiated component (phenotypically unrecognizable as to histogenesis) from an otherwise low-grade/borderline neoplasm, with well-defined morphologic demarcations between the aforementioned areas and a relatively worsened clinical course. The phenomenon of tumor dedifferentiation has subsequently been described in a wide variety of bone, soft tissue, and epithelial and neural neoplasms [2].

Tumor dedifferentiation is probably most well characterized in lipogenic tumors. Although it had been previously noted that well-differentiated and pleomorphic sarcomas may be admixed [3, 4], dedifferentiated liposarcoma (DDLS) was formally described by Evans in 1979 [5]. In a series of 55 liposarcomas, 8 cases (7 retroperitoneal, 1 spermatic cord) were noted to display “distinct areas of well-differentiated liposarcoma and of cellular spindle cell or pleomorphic sarcoma without recognizable lipogenesis” [5]. None of the latter cases displayed a mitotic count of >5/10 high power fields, and the cellular areas were often sharply demarcated from the well-differentiated areas [5]. Subsequent reports indicated that (a) dedifferentiation in well-DDLS is probably not anatomic site specific but time dependent [6], (b) most retroperitoneal tumors previously classified as malignant fibrous histiocytomas are actually DDLS [7], (c) most examples of DDLS present as de novo lesions rather than as a complication of a preexisting well-differentiated liposarcoma [8], (d) DDLS may be classified into high and low grade depending on mitotic activity and cellularity, although the prognostic significance of this gradation is unclear [8–11].

As with their clinical spectrum, the pathologic spectrum of DDLS has been similarly expanded. Newer morphologic observations have included a potentially poorly defined interface between the DDLS and its well-differentiated component [8], whorled, meningothelial-like structures [8, 12–14], a prominent inflammatory component [9], a micronodular growth pattern [9], a hemangiopericytomatous vascular pattern [8, 9], and heterologous differentiation [8, 9, 14–20].

Chondroid metaplasia, rhabdomyosarcomatous, and smooth muscular differentiation have been described in well-differentiated and myxoid liposarcomas [2, 21–24]; the term *lipoleiomyosarcoma* is commonly used for morphologic admixtures of well-differentiated liposarcoma and leiomyosarcoma. [23]. However, the probability for histologic misclassification is highest when high-grade DDLS displays heterologous differentiation [4, 5, 8, 9, 11–16]. Lines of heterologous differentiation that have been described in DDLS include leiomyosarcomatous, osteochondrosarcomatous, angiosarcomatous, and rhabdomyosarcomatous and do not appear to have independent prognostic significance [4, 5, 8, 9, 11–16, 25]

This report describes an example of a retroperitoneal DDLS with extensive leiomyosarcomatous differentiation that was accompanied by high levels of serum beta-human chorionic gonadotropin (*β*-hCG). This composite of findings has, to our knowledge, not been previously reported.

## 2. CLINICAL HISTORY

In 2006, a 67-year-old Caucasian female presented with episodic right upper quadrant abdominal pain and associated nausea. Her past medical history was significant for an infiltrating ductal breast carcinoma (treated with radical mastectomy followed by 4 cycles of adriamycin in 1999), a stable plasma cell dyscrasia (multiple myeloma diagnosed by a bone marrow biopsy and serum protein electrophoresis in 2003, treated with high dose chemotherapy, and which was associated with a mild normocytic anemia in the ensuing period), and a hysterectomy/salpingo-oophorectomy (for benign indications). A computed tomographic (CT) scan of the abdomen showed a heterogeneously enhancing mass extending from the inferior right renal pole exophytically, with significant associated inflammatory changes and some peripheral coarse calcifications. Portocaval and mesenteric adenopathy were also present. Serum levels of alpha-fetoprotein, carcinoembryonic antigen, CA15-3, and CA27-29 were within normal limits. Notably, a serum *β*-hCG, requested inadvertently, measured 210.3 mIU/ml (reference value for nongestational adults <5 mIU/L).

Given the high suspicion for a renal cell carcinoma, the right kidney was resected as well as all grossly identifiable perinephric tumor. Intraoperatively, the mass was noted to be prominently adhered to the surrounding retroperitoneal tissue. A follow-up serum *β*-hCG, which was measured 26 days after the first measurement and 25 days after her surgical procedure, measured 1046 mIU/ml. An abdominal CT scan performed shortly thereafter revealed multiple hypodense liver masses measuring up to 5 cm, with an increase in the aforementioned portocaval adenopathy. Biopsy of one of the hepatic masses confirmed the presence of metastatic disease. The patient declined further therapeutic measures and expired 2 months later. An autopsy was not performed.

## 3. MATERIALS AND METHODS

Twenty sections of the retroperitoneal mass were routinely processed for microscopic evaluation. The liver biopsy was similarly processed. For immunohistochemistry, 4-5 *μ*-thick sections were cut and mounted on a glass slide, deparaffinized and rehydrated. Appropriate negative and positive controls were included. All assays were carried out in an Axiom 36 autostainer (Lab Vision Corporation, Fremont, Calif, USA). The following markers were evaluated in the liver biopsy and a representative section of the retroperitoneal mass, in parallel: *β*-hCG (clone CG04+CG05, prediluted, Lab Vision), keratin cocktail comprised of clones AE1/AE3 (Signet Corporation, Dedham, Mass, USA) and LP34 (DakoCytomation, Carpinteria, Calif, USA), dilution 1 : 200, Smooth muscle actin: SMA (clone1A4, prediluted, Lab Vision), Estrogen receptor-alpha: ER-alpha (clone ID5, dilution 1 : 50, DakoCytomation), Progesterone receptor: PR (clone PgR 636, prediluted, DakoCytomation), Desmin (clone D33, prediluted, Lab Vision), Epithelial membrane antigen (EMA) (clone E29, prediluted, ThermoFisher Scientific, Fremont, Calif, USA), MDM2 (clone IF2, dilution 1 : 100, Lab Vision), and placental-like alkaline phosphatase: PLAP (Clone SP15, prediluted, Lab Vision).

## 4. PATHOLOGIC FEATURES

### 4.1. Macroscopic

The kidney and associated perinephric tissue measured in aggregate 21 × 10 × 7 cm. Sectioning revealed an inferior pole mass measuring 9.5 cm in maximum dimension, which compressed and distorted the renal pelvis and the tissue in this region. The cut surface of the mass was largely firm and solid, tan-white in color, with hemorrhagic areas but no grossly identifiable necrosis.

### 4.2. Morphologic features

**Figure 1:**
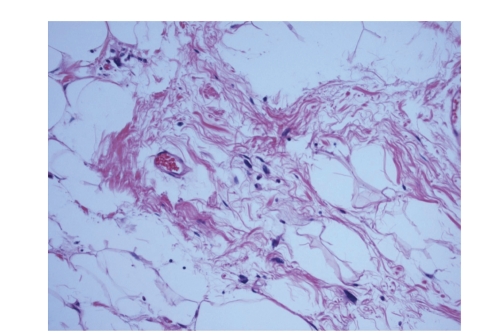
Well-differentiated component of the liposarcoma, showing atypical cells with nucleomegaly and hyperchromasia scattered within mature adipocytes and intervening fibrous septae.

**Figure 2:**
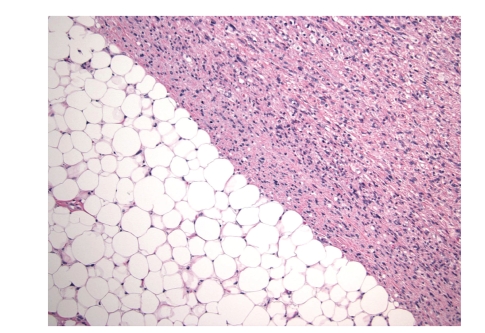
The interface between the well-differentiated and dedifferentiated components.

**Figure 3:**
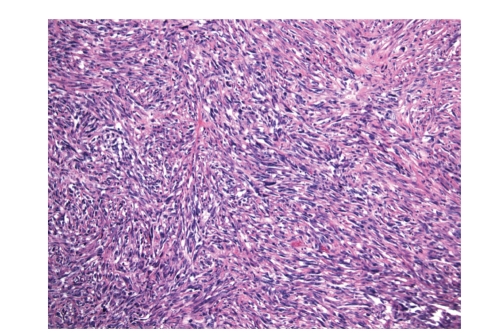
Cellular areas of the dedifferentiated component.

**Figure 4:**
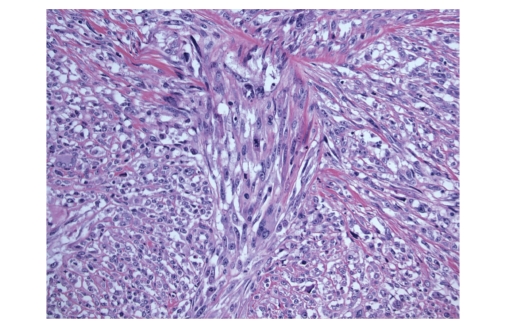
Some areas of the dedifferentiated component were comprised of cells with varying amounts of eosinophilic cytoplasm.

The retroperitoneal tumor displayed the characteristic morphologic features of DDLS. In the well-differentiated component, large coarse fibrotic bands were observed irregularly, coursing through lobules of well-differentiated adipocytes. Within the fibrous bands, the perivascular regions and within the adipocytic lobules were scattered cells with large irregular and hyperchromatic nuclei (Figure 1). The mitotic index in these regions was low. The dedifferentiated component comprised approximately 80% of the tumor mass. The interface between the well-differentiated and dedifferentiated components was variable: the interface was well demarcated in some regions (Figure 2), and highly irregular/infiltrative in others. The central portion of the mass was necrotic, while the surrounding areas, comprising approximately 10% of the mass were hypocellular and hyalinized. However, atypical cells well still recognizable in these regions. The remainder of the mass displayed a spectrum of pathologic changes with respect to cellularity, mitotic activity and atypia. Nonetheless, the tumor was predominantly a high-grade pleomorphic spindle cell sarcoma. In the most hypocellular regions, scattered atypical cells were clearly evident in a hyalinized background and displayed a maximum mitotic index of 11 MF/10 high power fields. Other areas were significantly more cellular, and were comprised of cells with hyperchromatic nuclei arranged in vague fascicles (Figure 3). These areas displayed the highest mitotic activity (maximum 36 MF/10 high power fields). Constituent cell displayed variable amounts of eosinophilic cytoplasm (Figure 4). The growth pattern of the tumor relative to the adjacent kidney was largely to displace it. However, there were areas where the tumor appeared to infiltrate in an interstitial pattern, with glomeruli encircled by tumor cells. The portions of the tumor not comprised of spindle cells were comprised of nondescript pleomorphic cells. Lipoblasts were evident throughout the tumor and there were no morphologically-recognizable syncytiotrophoblasts. The portion of the liver mass that was sampled was comprised predominantly of spindle cells as described above.

### 4.3. Immunophenotypic features

**Figure 5:**
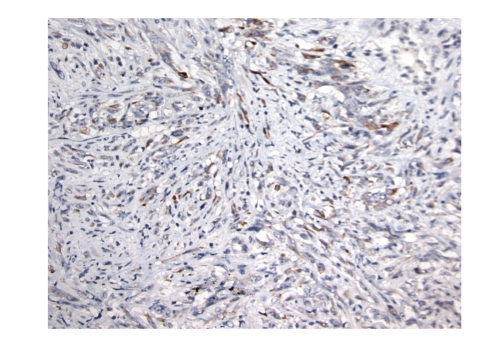
The tumor cells displayed diffuse immunoreactivity for *β*-hCG.

**Figure 6:**
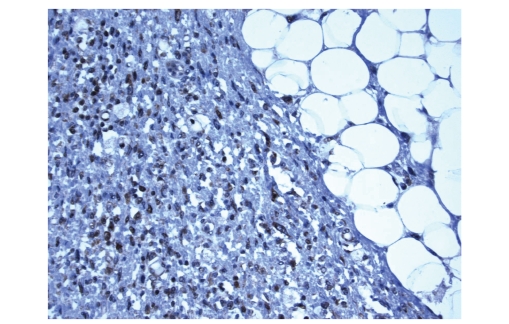
The tumor cells displayed diffuse immunoreactivity for MDM2.

The retroperitoneal tumor and liver mass displayed an identical immunoprofile, consistent with the latter being a metastatic deposit from the former. Both displayed diffuse immunoreactivity for desmin, SMA, *β*-hCG (Figure 5), and MDM2 (Figure 6). The tumors were negative for ER, PR, EMA, PLAP, and keratins.

## 5. DISCUSSION

hCG, a glycoprotein with a molecular weight of 38 000, is comprised of two subunits—*α* and *β*—that are noncovalently bound to form a heterodimer [26, 27]. The *α* subunit of hCG is identical to the *α* subunits of luteining hormone, follicle stimulating hormone, and thyrotropin; the *β* subunit therefore provides hormonal specificity [26, 27]. The measurement of serum *β*-hCG has its greatest clinical utility in the diagnosis of gestation, congenital disorders, and germ cell neoplasia. However, it has long been recognized that a wide variety of other nongerm cell neoplasms, predominantly carcinomas, may be associated with elevated serum *β*-hCG, in which it appears to function as an autocrine growth factor by inhibiting apoptosis [28, 29]. The expression of hCG has also been found to be an adverse prognostic factor in some specific histotypes such as small cell lung carcinoma, transitional cell carcinoma of the urinary bladder, and prostatic and colorectal adenocarcinoma [30–33]. Rare examples of osteosarcoma [28, 34–36], chondrosarcoma [37], and leiomyosarcoma [38–43] have been associated with elevated serum *β*-hCG. Amongst the six previously reported examples of *β*-hCG-producing leiomyosarcomas, two originated in the retroperitoneum [42, 43], two in the spermatic cord [39, 41], one in the uterus [40], and one in the small intestine [38]. Notably, five of the six patients succumbed to their disease within one year of their diagnoses. However, the sarcomas were high grade; thus the independent prognostic significance of *β*-hCG remains unclear.

In the present case, a DDLS with extensive leiomyosarcomatous differentiation was associated with *β*-hCG production, as evidenced by demonstration of *β*-hCG expression in the tumor and its metastatic deposit by immunohistochemistry, and an elevation of serum *β*-hCG that was temporally related to tumor progression. The diagnostic utility of MDM2, a recently described marker reportedly useful for distinguishing DDLS from their histologic mimics [44], was confirmed, as this marker helped to establish, in addition to morphologic features, underlying lipogenic differentation.

*β*-hCG production by DDLS has not been previously reported to our knowledge and is presumed to represent “aberrant” differentiation in a high-grade tumor. Nonetheless, this finding expands the clinicopathologic spectrum of lipogenic tumors in general. Furthermore, it suggests that liposarcoma should be a differential consideration when elevated serum *β*-hCG is associated with a radiographic mass lesion, especially when the mass is retroperitoneal.
